# Cytosolic N-terminal arginine-based signals together with a luminal signal target a type II membrane protein to the plant ER

**DOI:** 10.1186/1471-2229-9-144

**Published:** 2009-12-08

**Authors:** Aurélia Boulaflous, Claude Saint-Jore-Dupas, Marie-Carmen Herranz-Gordo, Sophie Pagny-Salehabadi, Carole Plasson, Frédéric Garidou, Marie-Christine Kiefer-Meyer, Christophe Ritzenthaler, Loïc Faye, Véronique Gomord

**Affiliations:** 1 Laboratoire GLYCAD, IFRMP 23, Université de Rouen, 76821 Mont Saint Aignan Cedex, France; 2 Institut de Biologie Moléculaire des plantes, 12 rue du Général Zimmer, 67084 Strasbourg Cedex, France

## Abstract

**Background:**

In eukaryotic cells, the membrane compartments that constitute the exocytic pathway are traversed by a constant flow of lipids and proteins. This is particularly true for the endoplasmic reticulum (ER), the main "gateway of the secretory pathway", where biosynthesis of sterols, lipids, membrane-bound and soluble proteins, and glycoproteins occurs. Maintenance of the resident proteins in this compartment implies they have to be distinguished from the secretory cargo. To this end, they must possess specific ER localization determinants to prevent their exit from the ER, and/or to interact with receptors responsible for their retrieval from the Golgi apparatus. Very few information is available about the signal(s) involved in the retention of membrane type II protein in the ER but it is generally accepted that sorting of ER type II cargo membrane proteins depends on motifs mainly located in their cytosolic tails.

**Results:**

Here, using *Arabidopsis *glucosidase I as a model, we have identified two types of signals sufficient for the location of a type II membrane protein in the ER. A first signal is located in the luminal domain, while a second signal corresponds to a short amino acid sequence located in the cytosolic tail of the membrane protein. The cytosolic tail contains at its N-terminal end four arginine residues constitutive of three di-arginine motifs (RR, RXR or RXXR) independently sufficient to confer ER localization. Interestingly, when only one di-arginine motif is present, fusion proteins are located both in the ER and in mobile punctate structures, distinct but close to Golgi bodies. Soluble and membrane ER protein markers are excluded from these punctate structures, which also do not colocalize with an ER-exit-site marker. It is hypothesized they correspond to sites involved in Golgi to ER retrotransport.

**Conclusion:**

Altogether, these results clearly show that cytosolic and luminal signals responsible for ER retention could coexist in a same type II membrane protein. These data also suggest that both retrieval and retention mechanisms govern protein residency in the ER membrane. We hypothesized that mobile punctate structures not yet described at the ER/Golgi interface and tentatively named GERES, could be involved in retrieval mechanisms from the Golgi to the ER.

## Background

In eukaryotic cells, the membrane compartments that constitute of the exocytic pathway are traversed by a constant flow of lipids and proteins. This is particularly true for the endoplasmic reticulum (ER), the main "gateway of the secretory pathway" [1], where biosynthesis of sterols, lipids, membrane-bound and soluble proteins, and glycoproteins occurs. Maintenance of the resident proteins in this compartment implies they have to be distinguished from the secretory cargo. To this end, they must possess specific ER localization determinants to prevent their exit from the ER, and/or to interact with receptors responsible for their retrieval from the Golgi apparatus. The tetrapeptide H/KDEL is the best characterized signal contributing to the accumulation of most soluble protein in the ER lumen [2-6]. Specific recognition of this tetrapeptide sequence by the ERD2-like receptor, in post-ER compartments, initiates the formation of COPI-coated vesicles, which transport the H/KDEL-containing soluble proteins selectively from the Golgi back to the ER [7-9].

Retrieval mechanisms from the Golgi to the ER are also responsible for ER location of some type I and II transmembrane proteins, in animals cells by interaction with subunits of the COPI machinery [8,10] (see Additional file 1 for membrane protein topology). Indeed, sorting of ER membrane residents depends on the specific interaction of motifs mainly located in their cytoplasmic tails. For instance, many type I membrane proteins located in the ER bear a di-lysine motif (K(X)KXX) in their C-terminal cytosolic tail [11]. In addition, the efficiency of a di-lysine motif for ER localisation of transmembrane proteins in cells has also been described in mammals, yeasts and plants [12-15], suggesting a conservation of the machinery. The di-lysine motifs can either act as direct retention signals or through a retrieval mechanism from the Golgi often associated with the acquisition of Golgi-specific carbohydrate modifications [16-19]. Some sequence flexibility can be observed concerning the dibasic motif(s) [20], in particular, lysine residues within non-type I membrane proteins are sometimes substituted by arginine [12]. Moreover, the amino acids (aa) flanking the di-lysine motif are important; since serine or alanine residues generally favor efficient retention while the proximity of glycine or proline residues completely disrupts ER retention capacity [11]. Finally, di-lysine ER-retention/retrieval signals require a strict spacing relative to the C-terminus [12,21,22].

On the other hand, some ER-resident membrane proteins contain a di-arginine motif acting as a retention/retrieval signal in animal cells. This motif is made of either two consecutive arginine residues located at position 2-3, 3-4, 4-5 with respect to the N-terminus of the protein or of arginine residues separated by an amino acid and located at position 2-4, 3-5. This motif was first described in yeast for signal-mediated retrieval of type II membrane proteins from the Golgi to the ER [23,24]. It is now generally admitted that di-arginine motifs are much more frequent than di-lysine motifs. They are found in a variety of cytosolic positions, including loops, at the C- and N- terminal end of type I and II membrane proteins respectively [25]. Like the di-lysine motif, the di-arginine motif efficiency is influenced by surrounding residues [26-28]. Structural analysis of N-linked glycans revealed a Golgi-to-ER retrograde transport mechanism for ER membrane glycoproteins containing a di-arginine motif indicating they act as ER retrieval signals as described for most di-lysine motifs [29].

Several other motifs have occasionally been described for ER retention of membrane proteins in eukaryotic cells. For instance the diphenylalanine (FF) motif, present in type I proteins of the p24 family, is essential for COPI coat protein interactions triggering Golgi to ER retrograde transport [30,31]. Similarly, Cosson et *al*. [32] identified a new COPI-binding motif containing a critical aromatic residue involved in ER retrieval.

In addition to retrieval mechanisms, the strict retention of ER-resident proteins has also been investigated. It was shown for Sec12p (a type II ER membrane protein), that the TMD is responsible for recycling whereas the cytosolic tail is involved in strict retention [33]. ER residency by direct retention can be also accomplished by oligomerization of protein subunits into large complexes, *via *their transmembrane and/or the luminal domains [29,34-38]. It is important to note that both mechanisms, retention and retrieval, are not exclusive and can function either in parallel or in combination [29].

In plants, few molecular signals responsible for protein residency in the ER have been described [39]. For soluble proteins, K/HDEL is largely predominant [3-5]. For type I membrane proteins, signals include C-terminal di-lysine motifs [13,14,40], the aromatic aa-enriched ER retrieval signal [14] and the length of the TMD [41]. To our knowledge, so far, no information is available concerning signals responsible for type II membrane protein residency in the plant ER.

Alpha-glucosidase I is the first enzyme involved in the N-glycan maturation. This glycosidase removes the distal α-1,2-linked glucose residue from the oligosaccharide precursor, just after its transfer "en bloc" on the nascent protein. The function and consequently the location of this type II membrane protein in the ER is essential for plant development [42,43].

In a previous study, we have shown that *A. thaliana *glucosidase I (*At*GCSI) is located exclusively in the ER [44]. This localization is consistent with a trimming of the first glucose residue from the precursor oligosaccharide. Here, the analysis of the N-terminus of this glycosidase has allowed the identification of two independent types of signals conferring ER residency to a type II membrane protein. Thus, di-arginine-based motifs initially located in the cytosolic face of *At*GCSI are sufficient to confer ER residency of a membrane reporter protein. As the presence of a second type of signal in the luminal part of *At*GCSI is also sufficient for ER retention, we propose that the arginine-based motifs may act as salvage signals to localize the full-length protein in this compartment.

## Results

### The cytosolic tail of *At*GCSI contains ER targeting information

The cytosolic region of many membrane proteins residing in the mammalian and yeast ER contains signals which facilitate either their strict retention in the ER [29,33-38,45] or their retrieval from the Golgi to the ER [11,29,46]. In plants, only very few studies refer to the characterization of cytosolic motifs responsible for membrane protein retention in the ER [13,14,31,40].

With the aim to identify a conserved ER targeting motif in the cytosolic tails of the different GCSI cloned so far, we aligned their sequences (Table 1). The size of GCSI cytosolic tail is very different from one species to another varying from 11 aa in *Neurospora crassa *to 62 aa in *Oriza sativa*. However, in each case, the cytosolic tail is very polar, arginine and lysine residues being largely represented. In particular, arginine blocks near the N-terminal end are identified in six out of twelve GCSI sequences. This block was shown to contain ER trafficking information in human GCSI [29].

**Table 1:** Comparison of the cytosolic tail sequence and transmembrane domain length of glucosidases I cloned from different species

Organism	Cytosolic tail sequence	TMD length
*Arabidopsis thaliana* AJ278990	MTGAS**RR**SA**R**G**R**I**K**SSSLSPGSDEGSAYPPSI**RR**G**K**G**K**ELVSIGAFKTNL**K**	18
*Oryza sativa* BAB86175.1	MSGGGGSSSV**RR**PVAAA**R**S**R**SGPEPDA**RR**AAAAAAAAAAAAA**RRR**G**R**GDHGPL**R**LMEVSPRN	23
*Neurospora crassa* CAC18158.1	MAPPPP**R**QP**R**Q	23
*Strongylocentrotus Purpuratus* XP_797552.1	>MAA**R**T**R**IADSGGGA**R**S**R**ET**K**T**K**P**K**SGNGAQS**R**NNETQSSS**K**N	23
*Danio rerio* *XP_696318.1*	MG**RRRKR**VATGDGVPSP**RK**EE**K**APAPP**RK**E**KKKK**TDIG**K**	24
*Apis melifera* XP_623340.1	MSILNISITVLCIAIATWFSY**K**GYLET**R**VNTPYDI**KK**LVTIS	23
*Tribolium castaneum* XP_972740.1	MA**R**Q**RR**TQGAADPN**K**GTNSSSSNGSNSTNN**R**SS**K**STS	23
*Enchytraeus japonensis* BAE93517.1	MA**KKK**VP**R**E**K**NHSGGTT**RR**TSESSSNNHADS**KR**QI**R**I**K**LNE**KRKR**QEPGS**K**	23
*Caenorhabditis elegans* NP_502053.1	MH**R**EHEEMHQPS**RRRR**PP**R**EVE**R**PSATI**R**YEPVAEPEPWCSFCSWD	23
*Homo sapiens* NP_006293.2	MA**R**GE**RRRR**AVPAEGV**R**TAE**R**AA**R**GGPG**RR**DG**R**GGGP**R**	21
	-60 -50 -40 -30 -20 -10 -1	

Numbers below the cytosolic tail sequences indicate the position from the transmembrane domain (TMD). Bold letters highlight the importance of arginine (R) and lysine (K) residues. Note underlined sequence from *H. sapiens *retains a reporter membrane protein in the ER in plant cell.

**Figure 1:**
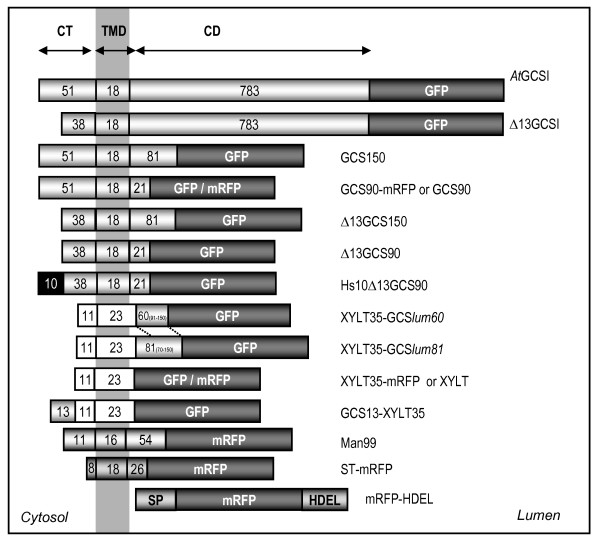
**Schematic representation of the fusion proteins analyzed in this study**. *At*GCSI: full-length *A. thaliana *α-glucosidase I fused to GFP. Δ13GCSI: GCSI minus the first 13 N-terminal aa (MTGASRRSARGRI-). GCS150: the first 150 aa of GCSI fused to GFP. GCS90: the first 90 aa of GCSI fused to GFP or mRFP. Δ13GCS150: GCS150 minus the first 13 N-terminal aa. Δ13GCS90: GCS90 minus the first 13 N-terminal aa. Hs10-Δ13GCS90: the first 10 N-terminal aa of *Homo sapiens *GCSI (MARGERRRRA-) fused at the N-terminus of Δ13GCS90. XYLT35: the first 35 aa of *A. thaliana *β-1,2-xylosyltransferase fused to GFP or mRFP [47]. XYLT35-GCS*lum60*: the first 35 aa of XYLT fused to the first 60 aa of the luminal domain of GCSI (Pro91 to Cys150) and to GFP. XYLT35-GCS*lum81*: the first 35 aa of XYLT fused to the first 81 aa of luminal domain of GCSI (Arg70 to Cys150) and to GFP. GCS13-XYLT35: the 13 first N-terminal aa of GCSI fused to XYLT35. ST-mRFP: the first 52 aa of a rat α-2,6-sialyltransferase (ST) fused to mRFP [90]. mRFP-HDEL: mRFP under the control of the sporamine signal peptide and the HDEL ER retention sequence. CT: cytosolic tail; TMD: transmembrane domain; CD: C-terminal domain.

**Figure 2:**
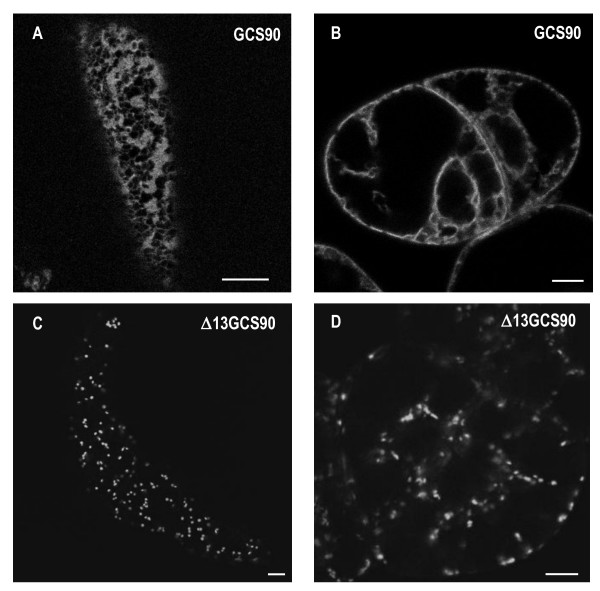
**The 13 first N-terminal amino acids of *At*GCSI contain ER targeting information**. CLSM analysis of transgenic tobacco BY-2 cells showing cortical views (**A**, **C**) or cross sections (**B**, **D**). (**A**, **B**) GCS90 accumulates in the ER in BY-2 suspension-cultured cells. (**C**, **D**) Δ13GCS90 accumulates into the Golgi apparatus. Bars = 8 μm.

*At*GCSI is an ER type II membrane protein, composed of a 51 aa cytosolic tail (CT), a 18 aa transmembrane domain (TMD) and a large 783 aa C-terminal domain (CD) oriented in the lumen of the ER and containing the catalytic site [42,44] (Figure 1). As illustrated (Figure 2AB), we have shown in a previous work that the first 90 aa (CT+TMD+ 30 aa of the stem) located at the N-terminal end of the *At*GCSI, are sufficient to retain a reporter protein in the ER [44]. The *At*GCSI cytosolic domain of 51 aa contains six arginine residues including four arginines located at position 6, 7, 10 and 12 and a doublet at the position 33,34 relative to the N-terminal end.

**Figure 3:**
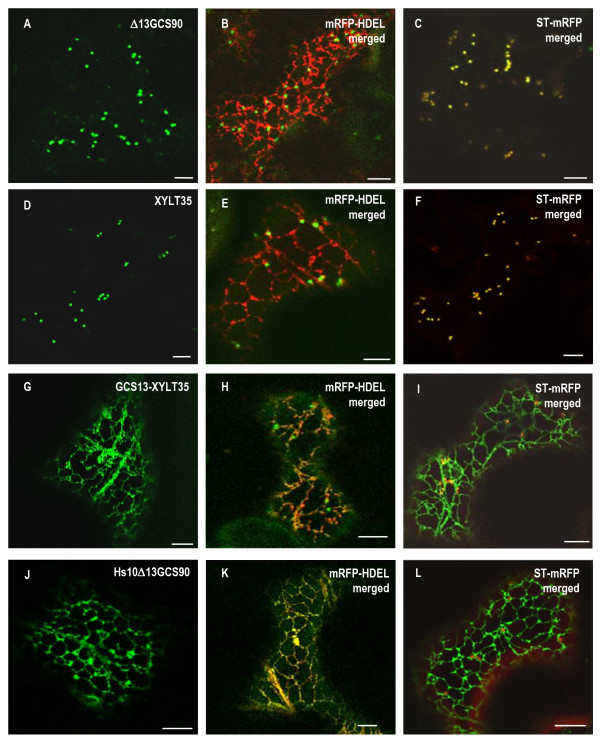
**Arginine-rich ER targeting sequences are conserved for GCSI between kingdoms**. CLSM analysis of *Nicotiana tabacum *leaf epidermal cells expressing GFP fusions alone (left panels), or co-expressing GFP fusions and either the ER marker mRFP-HDEL (middle panels), or the Golgi marker ST-mRFP (right panels). Δ13GCS90 (**A**-**C**) is exclusively located in the Golgi and perfectly co-localizes with ST-mRFP (**C**). XYLT35 is also located in the Golgi (**D**-**F**); [44]. When GCS13-XYLT35 (**G**) is co-expressed with mRFP-HDEL, the ER appears in yellow and the Golgi remains green (**H**) whereas when GCS13-XYLT35 is co-expressed with ST-mRFP the Golgi is yellow and the ER is green (**I**) showing GCS13-XYLT35 has a dual location in the ER and in the Golgi. Interestingly, when the first 13 N-terminal amino acids of GCS90 are replaced by the first 10 N-terminal amino acids of the human GCSI, Hs10Δ13GCS90 is located exclusively in the ER (**J**) as illustrated from colocalization with mRFP-HDEL (**K**) and the absence of overlap for GFP and RFP signals when it is co-expressed with ST-mRFP (**L**). This together with data presented **Table 1 **suggests that arginine-rich ER targeting sequences are conserved for GCSI between kingdoms. Bars = 8 μm.

To define more precisely the sequence in the cytosolic tail of *At*GCSI containing ER location information, the first 13 aa located at the N-terminal end of GCS90 were deleted and the resulting chimeric protein was named Δ13GCS90 (Figure 1). This truncation removed potential dibasic motifs RR or RXR that might function in ER localization [28], while others (RR or KXK) remained in the cytosolic tail of this fusion protein. When expressed in tobacco BY-2 cells or leaf epidermal cells, Δ13GCS90 was found into bright spots (Figure 2CD) that colocalized with the Golgi marker ST-mRFP (Figure 3A-C) [44] but no longer localized with the mRFP-HDEL ER marker (Figure 3B). These results indicate that the first 13 aa of *At*GCSI are required for GCS90 accumulation in the ER.

**Figure 4:**
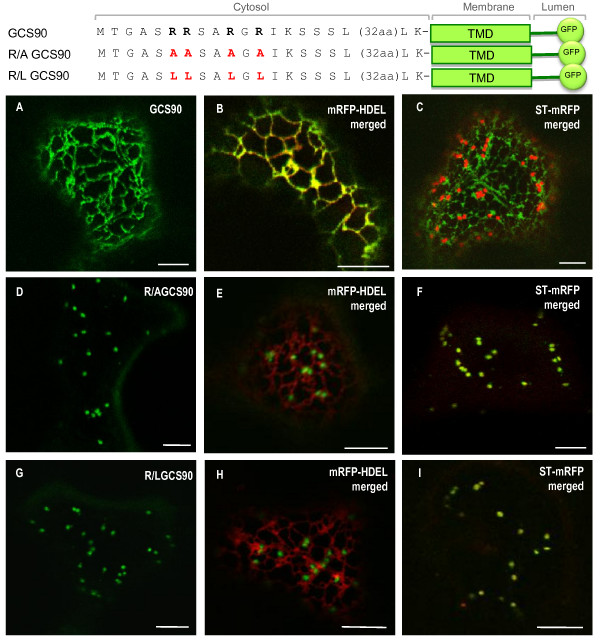
**The N-terminal arginine residues of *At*GCSI contain ER localization information**. CLSM analysis of *Nicotiana tabacum *leaf epidermal cells expressing GFP fusions alone (left panels), or co-expressing GFP fusions and the ER marker mRFP-HDEL (middle panels), or co-expressing GFP fusions together with the Golgi marker ST-mRFP (right panels). GCS90 (**A**) co-localizes with mRFP-HDEL (**B**, ER in yellow) but not with ST-mRFP (**C**, ER in green, Golgi in red). When the four arginine residues in position 6, 7, 10 and 12 are replaced by alanine or leucine residues, R/A GCS90 (**D**-**F**) or R/L GCS90 (**G**-**I**) accumulates exclusively in the Golgi showing that arginine residues are involved in *At*GCSI ER localization. Bars = 8 μm.

In order to determine whether this 13 aa peptide sequence affects the targeting a Golgi-resident membrane protein, it was fused to the Golgi marker XYLT35 to give GCS13-XYLT35 (Figure 1). As illustrated in figure 3D-F, XYLT35 resides exclusively in the Golgi apparatus and it was previously shown to preferentially accumulate in the medial Golgi [47]. In contrast, GCS13-XYLT35 was found as a bright network (Figure 3G-I) that colocalized with the mRFP-HDEL ER marker (Figure 3H) and was very similar to the pattern observed for GCS90 (compare to Figure 2AB). In addition to this strong ER labeling, a few bright spots were also occasionally observed when GCS13-XYLT35 was expressed (Figure 3H). These spots proved to be dynamic and colocalized partially with the late Golgi marker ST-mRFP (Figure 4I) indicating location in the early Golgi (Figure 3I), [44].

In conclusions, we show here that the first 13 aa of *At*GCSI are necessary to retain the GCS90 fusion protein in the ER and sufficient to relocate a medial Golgi marker mainly to the ER and to a lesser extent the early-Golgi.

### A cytosolic arginine-rich sequence is an ER targeting signal in plants

In order to further investigate whether another arginine-rich sequence could replace the 13 N-terminal aa of *At*GCSI responsible for ER retention, this peptide was replaced by the first 10 amino-terminal residues of human GCSI and the resulting fusion was named Hs10Δ13GCS90 (Figure 1). After transient expression in tobacco leaf epidermal cells, Hs10Δ13GCS90 localized in the ER (Figure 3J-L), thus demonstrating that the N-terminal arginine-rich cytosolic sequence of human GCSI is functional in plants. Similarly, the C-terminal arginine-rich cytosolic tail of *Arabidopsis *calnexin, a type I membrane protein changed the localization of the type II Δ13GCS90 from the Golgi to the ER (see Additional file 2)

### Arginine residues in the cytosolic tail of *At*GCSI contain ER localization information

In order to define whether arginine residues within the first 13 aa of GCS90 play a key role in ER targeting, these residues were first replaced by either leucine or alanine residues using site-directed mutagenesis (see Table 2 for the construct details) and the resulting fusion proteins were expressed in tobacco cells.

**Table 2:** Sub-cellular localization of GCS90 after arginine (R) substitutions in the cytosolic tail.

Mutants	Cytosolic domain	Sub-cellular localization
	6 7 10 12	
GCS90	M T G A S **R R **S A **R **G **R **I K S S S L-32aa	ER
Δ13GCS90	M K S S S L-32aa	Golgi

Hs10Δ13GCS90	M A R G E **R R R R **A K S S S L-32aa	ER
GCS13-XYLT35	M T A G A S **R R **S A **R **G **R **I-10aa	ER + GA
CNX11-XYLT35	M N D **R R **P Q **R **K **R **P A-10aa	ER + GA

R/L_6-7_GCS90	M T G A S L L S A **R **G **R **I K S S S L-32aa	ER + punctate structures
R/L_10-12_GCS90	M T G A S **R R **S A L G L I K S S S L-32aa	ER + punctate structures
R/L_6-12_GCS90	M T G A S L **R **S A **R **G L I K S S S L-32aa	ER + punctate structures
R/L_6-10_GCS90	M T G A S L **R **S A L G **R **I K S S S L-32aa	ER +GA
R/L_7-12_GCS90	M T G A S **R **L S A **R **G L I K S S S L-32aa	Golgi +ER
R/L_7-10_GCS90	M T G A S **R **L S A L G **R **I K S S S L-32aa	Golgi
R/AGCS90	M T G A S A A S A A G A I K S S S L-32aa	Golgi
R/LGCS90	M T G A S L L S A L G L I K S S S L-32aa	Golgi

GCS90 is exclusively located in the ER (Figure 4A) and perfectly co-localizes with the ER marker mRFP-HDEL (Figure 4B), but not with the late Golgi marker ST-mRFP (Figure 4C). When arginine residues, in position 6, 7 10 and 12 (R_6_, R_7_, R_10 _and R_12_, respectively) were all replaced by alanine residues, GCS90 mutant (R/AGCS90) was found to accumulate exclusively in the Golgi apparatus as illustrated from its co-localization with ST-mRFP (Figure 4D-F). The same effects on sub-cellular localization were observed for R/L GCS90 after substitution of the four arginine residues by leucines, (Figure 4G-I). These observations indicate that four arginines in position 6-7-10 and 12 present in the cytosolic tail of *At*GCSI encode information necessary for ER residency of membrane reporter protein.

**Figure 5:**
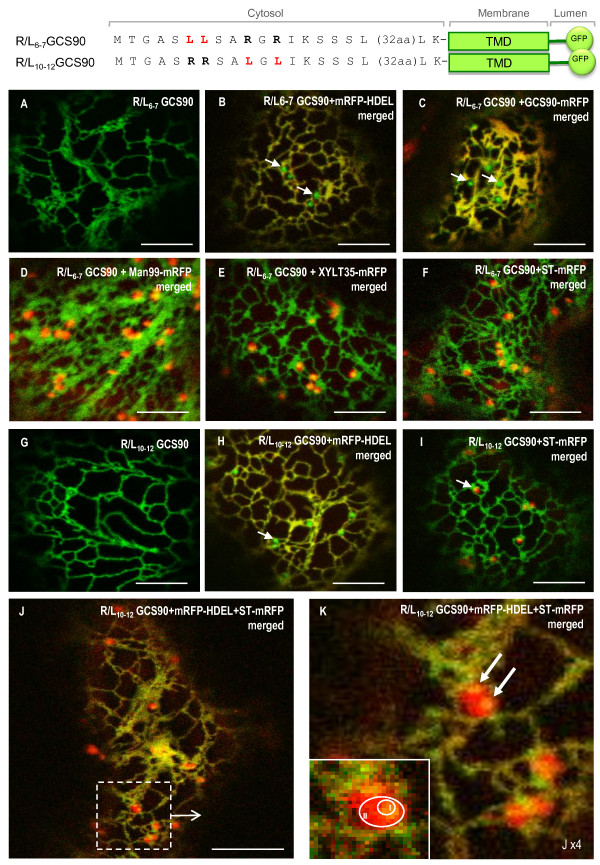
**Punctate structures do not accumulate ER resident proteins and are distinct from Golgi stacks**. When arginine residues are mutated by pairs, R/L_6-7_GCS90 (**A**-**I**) and R/L_10-12_GCS90 (**G**-**K**) are located in the ER (**A**, **G**). Co-expression with soluble ER marker mRFP-HDEL (**B**, **H**) or membrane (**C**) ER marker GCS90-mRFP reveals those markers are excluded from the punctate structures that appear in green (arrows). Punctate structures are closely associated to Golgi stacks labelled with the *cis*-Golgi marker Man99-mRFP (**D**), the medial Golgi marker XYLT35-mRFP (**E**) or *trans*-Golgi marker ST-mRFP (**F**, **I**). When the constructs highlighting punctate structures are co-expressed together with the ER marker mRFP-HDEL and the Golgi marker ST-mRFP, the ER and the punctate structures appear in yellow (**J**). When zooming, micrograph suggests punctate structures can be closed to the ER (**K**, top and bottom arrows). Zone I corresponds to the co-localization area between a punctate structure and a Golgi whereas zone II corresponds to the Golgi only (**K**, insert). Arrows indicate the punctate structures.

To further dissect this cytosolic signal, an exhaustive pair-wise leucine scanning mutagenesis of all four arginine residues was performed and results related to the location of the mutants in tobacco leaf epidermal cells are summarized in Table 2. All mutations affected the localization of GCS90. Thus, R/L_6-7_GCS90 and R/L_10-12_GSC90 were found in the ER (Figure 5A, I) and in additional punctate structures (Figure 5B, H, arrows) that appear distinct from Golgi stacks (Figure 5D-F and 5I). Similar results were obtained for R/L_6-12_GCS90 (Additional file 3). Remarkably, the mRFP-HDEL soluble and the GSC90-mRFP membrane ER markers were excluded from these punctate structures (Figure 5B-C and 5H). Finally, Constructs in which mutated arginine residues were distant by more than two aa (R/L_6-10_; R/L_7-12_; R/L_7-10_) all displayed a strict Golgi (illustrated with R/L_7-10_, Additional file 3GH) or a dual Golgi-ER pattern (illustrated with R/L_6-10 _Additional file 3CD; or with R/L_7-12 _Additional file 3EF,). These findings indicate that a cytosolic RR or RXR or RXXR motif is sufficient to confer ER residency to a membrane reporter protein.

### Towards the characterization of punctate structures labeled after arginine substitution

Considering that fusion proteins harboring only one di-arginine motif: RR, RXR or RXXR accumulate in the ER and in punctate structures associated with the Golgi, the next challenge was to identify the nature of these fluorescent punctate structures from which the ER markers are excluded. Coexpression of R/L_10-12_GCS90 with an ER and a Golgi marker simultaneously, revealed that the punctate structures are closely associated but nevertheless distinct and smaller than Golgi stacks (Figure 5JK and insert). Interestingly, units formed by association of one dictyosome and one punctate structure move together along the ER and never dissociate (see Additional file 4). Considering these observations, we propose that punctate structures are small intermediate domains located between the ER and the Golgi, from which ER resident soluble or membrane proteins are excluded (Figure 5B and 5C).

Based on the observation that punctate structures are strongly associated with the Golgi and move together with the Golgi stacks along the ER cortical network, we speculated first that they could correspond to ER-exit-sites (ERES) initially described by daSilva et *al*. [48].

**Figure 6:**
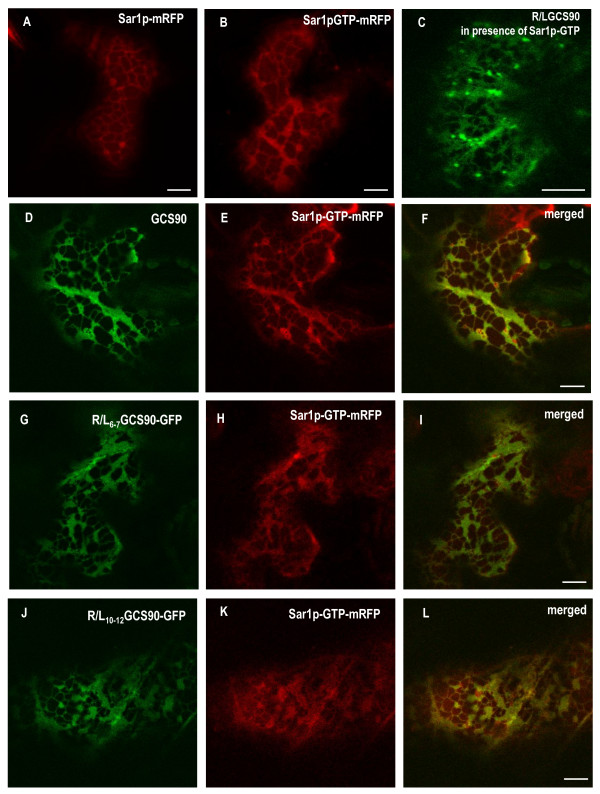
**Sar1p-GTP regulates ER to Golgi traffic of GCS90 and induces the disappearance of the punctate structures**. CLSM analysis of *Nicotiana tabacum *leaf epidermal cells expressing GFP-fusions simultaneously with Sar1p variants. Sar1p-mRFP (**A**) and Sar1p-GTP-mRFP (**B**) are accumulated at the ER. Because Sar1p-GTP-mRFP blocks ER exit, membrane proteins accumulate in the ER and the ER membrane morphology turns into fenestrated sheets (**B**). In presence of Sar1p-GTP-mRFP, the Golgi fusion R/LGCS90 is blocked in the ER (**C**, compare with pattern presented **Figure 5G**). When GCS90 (**D**-**F**), R/L_6-7_GCS90 (**G**-**I**) or R/L_10-12_GCS90(**J**-**L**) are co-expressed with Sar1p-GTP-mRFP, the expression patterns remain unchanged, (compare to **Figure 7G-I, A-C **and **D-F**, respectively), except that the punctate structures have disappeared. Bars = 8 μm.

It was previously shown that a GTP-locked form of Sar1p accumulates to ERES [48] and exerts a dominant negative effect on protein secretion [48-52]. When Sar1p-mRFP or Sar1p-GTP-mRFP were expressed alone, they were both located to the cytoplasm and to the ER (Figure 6AB, **respectively**) but the ER morphology was different. Indeed, Sar1p GTP blocking ER exit, R/LGCS90 was found in the ER and in the Golgi when expressed together with the GTP-locked form of Sar1p (Figure 6C), and, as a consequence, the ER membranes turned into a lamellar sheet. In addition, Sar1p-GTP-mRFP and GCS90 perfectly co-localised (Figure 6D-F). To test if the small punctate structures were sensitive to an ER exit blockage, R/L_6-7_GCS90 and R/L_10-12_GCS90 were co-expressed with Sar1p-GTP-mRFP (Figure 6G-I and 6J-L). Interestingly, no punctate structures were observed showing that the presence of punctate structures depends on active COPII machinery.

**Figure 7:**
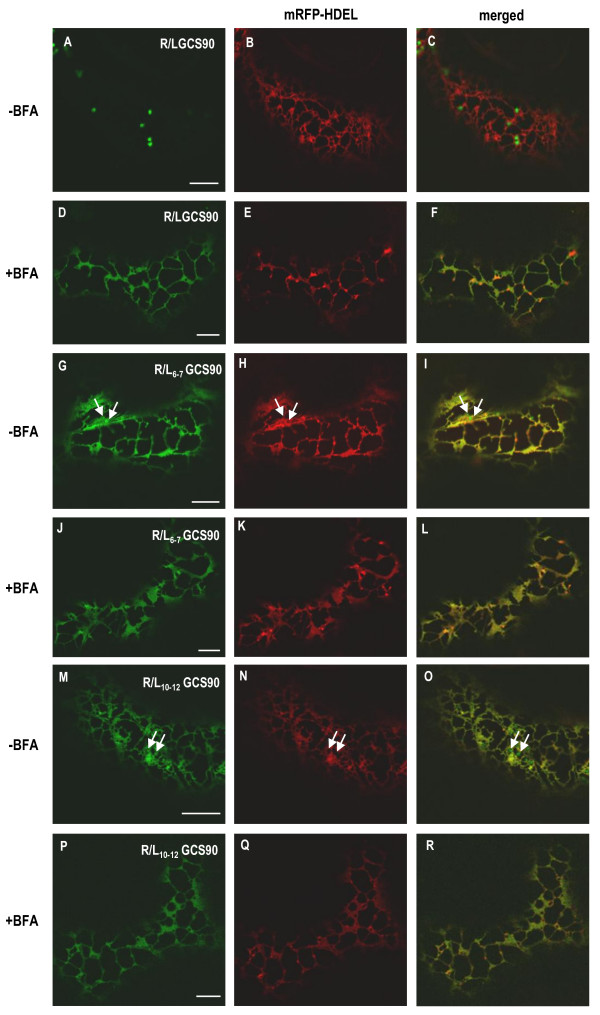
**Punctate structures diappear when COPI-mediated retrograde transport is inhibited with BFA**. CLSM analysis of *Nicotiana tabacum *leaf epidermal cells co-expressing GFP-fusions and mRFP-HDEL. Control cells co-expressing R/LGCS90 and mRFP-HDEL show green Golgi stacks and a red ER (-BFA, panels **A**-**C**). When cells are treated with BFA for 2 h, Golgi membranes are reabsorbed in the ER and the ER appears in yellow (+BFA, panels **D**-**F**). When cells co-expressing R/L_6-7_GCS90 (**G**-**L**) or R/L_10-12_GCS90 (**M**-**R**) and mRFP-HDEL are treated with BFA for 2 h, punctate structures disappear (**J**-**L **and **P**-**R **respectively).

The drug BFA blocks COPI-mediated retrograde transport. Thus, if the punctate structures were sensitive to BFA, this would suggest they are likely to be involved in retrograde Golgi to ER traffic. To test this hypothesis, cells co-expressing R/L_6-7_GCS90 or R/L_10-12_GCS90 and mRFP-HDEL were incubated for 2 h in the presence of BFA (Figure 7G-I and 7M-O respectively). In both cases, the ER turned into a lamellar pattern and the punctate structures disappeared (Figure 7J-L and 7P-R). As a control, we have observed BFA-induced redistribution of R/LGCS90 in the ER (Figure 7A-F). Together, these results indicate that inhibition of COPI-mediated retrograde transport by BFA abolishes the formation of punctate structures.

In conclusion, different GCS90 mutants harboring only one RR, RXR or RXXR motif accumulate in the ER and in punctate structures that do not contain ER soluble or membrane resident proteins, move together with the Golgi, but are not formed in the presence of Sar1p-GTP and disappear in the presence of BFA. Based on these results, our hypothesis is that these punctate structures could be involved in Golgi to ER retrograde transport.

### A luminal sequence in *At*GCSI also contains ER retention information

We have shown above that cytosolic arginine-motifs are sufficient to confer ER-residency to a Golgi reporter protein and their removal changes the localization of GCS90 from the ER to the Golgi. However, we observed that the deletion of the first N-terminal 13 aa from the full-length sequence of *At*GCSI (Δ13GCSI- Figure 1), does not modify the location of the *At*GCSI. The accumulation of Δ13GCSI in the ER shows that the arginine motifs are not necessary for ER residency of the full-length *At*GCSI protein and suggests that other ER retention signals must exist.

**Figure 8:**
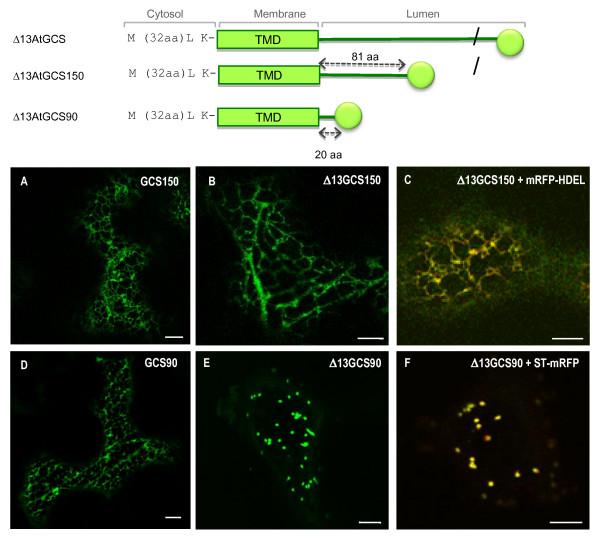
**The N-terminal arginine motifs are not the unique determinants responsible for ER retention of *At*GCSI**. When expressed in *Nicotiana tabacum *leaf epidermal cells Δ13GCS150-GFP is located in the ER (**B**) A as it was observed for the GCS150 (**A**) and confirmed after co-expression with the ER marker mRFP-HDEL (**C**). In contrast, Δ13GCS90-GFP is targeted to the Golgi apparatus (**E**) where it colocalizes with the Golgi marker ST-mRFP (**D**) whereas GCS90 is accumulated in the ER (**D**). Bars: 8 μm.

After successive deletion at the C-terminal end of Δ13GCSI, we have shown that, in contrast with the Golgi location of Δ13GCS90, the Δ13GSC150 containing the first 150 aa of At GCSI minus the first 13 aa (Δ13CT+TMD+81 aa of the stem) is detected exclusively in the ER (Figure 8A). In order to identify the sequence responsible for ER localisation of Δ13GCSI, the first 13 aa of the GCS150 were deleted and the resulting fusion protein (Δ13GCS150) was expressed *in N. tabacum *leaf epidermal cells, where it was found exclusively in the ER (Figures 1 and 8B), and perfectly co-localized with mRFP-HDEL (Figure 8C). In contrast, in the same conditions, Δ13GCS90 was detected exclusively in the Golgi apparatus (Figure 8EF). ER-specific targeting information is therefore contained within the *At*GCSI luminal domain, between the Pro 91 and Cys150.

**Figure 9:**
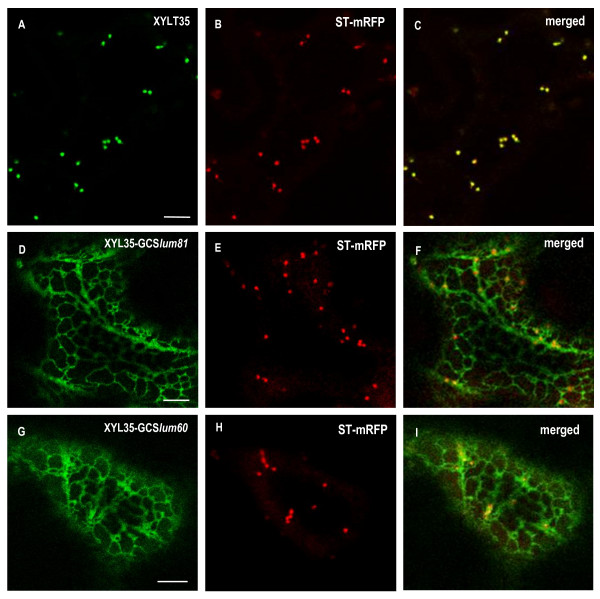
**A luminal domain of *At*GCSI is sufficient for targeting a Golgi marker into the ER**. (**A**-**C**) XYLT35 and ST-mRFP are targeted to the Golgi when expressed in *Nicotiana tabacum *leaf epidermal cells. (**D**-**F**, **G**-**I**) XYLT35-GCS*lum81 *or XYLT35-GCS*lum60 *where co-expressed with ST-mRFP. Both fusion proteins containing a 81 or a 60 aa long luminal domain of *At*GCSI fused to the Golgi marker XYLT35 (XYLT35-GCS*lum81 *and XYLT35-GCS*lum60 *respectively) are targeted to the ER. Bars = 8 μm.

To further investigate the ER targeting capacity of its luminal domain, an 81 aa long peptide corresponding to the N-terminal part of *At*GCSI luminal domain (from Arg70 to Cys150) was fused at the C-terminal end of the medial-Golgi marker XYLT35 (Figure 9D-F, and the resulting fusion protein was named XYLT35-GCS*lum81*). A shorter 60 aa peptide corresponding to the luminal domain of *At*GCSI from Pro91 to Cys150, was fused to XYLT35 to generate XYLT35-GCS*lum60 *(Figure 1). Both fusions were expressed in tobacco leaf epidermal cells. In agreement with its medial-Golgi localization, XYLT35 accumulated specifically in the Golgi (Figure 9A-C), whereas both XYLT35-GCS*lum81 *and XYLT35-GCS*lum60*, where detected in the ER (Figure 9D-F, G-I). Therefore, in addition to arginine-based motifs in its cytosolic tail, *At*GCSI contains additional information in its luminal domain from residues Pro91 and Cys 150 that is sufficient to confer ER localization.

## Discussion

Introduction of soluble or type I membrane proteins in the ER, is mediated by a cleavable N-terminal signal peptide. Then, ER protein localization is governed by different signals and mechanisms. It is well documented that soluble ER-resident proteins bear at their C-terminal end a H/KDEL tetrapeptide that ensure their retrieval from the Golgi apparatus to the ER when they escape to this organelle [3,5,9]*via *the binding to a receptor named ERD2-like [53-56] located throughout Golgi and in the ER [57,58]. In contrast, molecular signals responsible for the targeting of type I membrane proteins in the ER are not so well understood, especially in plant cells. For instance, a 17 aa TMD derived from human lysosomal protein LAMP1 was shown to mediate retention of GFP in the ER [41]. In addition, C-terminal dilysine motifs confer ER localization to type I membrane proteins [13,31,40]. Finally, a C-terminal ΦXXK/R/D/EΦ motif (where Φ is a large hydrophobic aa residue) is necessary and sufficient for the localization of type III membrane Δ^12 ^oleate desaturase FAD2 to the ER [14].

For type II membrane proteins, the TMD acts as a non-cleavable signal sequence (Additional file 1) and we have recently shown that in plant cell, the 16 aa TMD of soybean mannosidase I (ManI) is sufficient to retain GFP in the ER and the *cis*-Golgi whereas the 18 aa TMD of *At*GCSI is not responsible for the residency of this glucosidase in the ER [44]. Here we investigated the signals that mediate ER localization of *At*GCSI, a type II membrane enzyme playing a key role in seed development, as shown by characterization of the GCSI *Arabidopsis *mutant which produces shrunken seeds where embryo development is blocked at the heart stage [42].

### A cytosolic di-arginine motifs is sufficient for ER residency of a type II membrane protein

Based on the demonstration that the 13 first N-terminal aa of *At*GCS1 cytosolic sequence contain ER targeting information (Figure 3), we have substituted the four arginine residues in the sequence MTAGASRRSARGRI- with alanine or leucine residues. This mutation completely abolishes the ER retention capacity of this sequence, as R/LGCS90 and R/AGCS90 were found in the Golgi, thus demonstrating the key role of arginine residues. In addition, this 13 aa peptide was sufficient to relocalize, the medial Golgi marker XYLT35 to the ER when fused at its N-terminal end. A competition between the di-arginine motifs mediating ER localization and the TMD length of XYLT35 (23 aa), more consistent with a Golgi location, could explain why part of GCS13-XYLT35 is also detected in the Golgi apparatus.

In order to identify the minimal requirement for the ER targeting motif, the four arginine residues were mutated in pairs and it was found that two arginine residues organized as RR, RXR or RXXR motif were sufficient to confer ER residency. Consequently, three distinct di-arginine motifs sufficient for ER retention co-exist in the cytosolic tail of GCSI. In mammalian cells, N-terminal arginine residues were also shown to serve as ER signals for some type II membrane proteins [28,29]. For instance, the first 16 aa of human Iip33 (MH**RRR**S**R**SC**R**EDQKPV-) target not only Iip33 but also other type II membrane proteins to the ER and the minimal requirement for efficiency of this sequence is the presence of a diarginine RR or RXR motif [28]. On the other hand, in the first 10 aa of human GCSI (MA**R**GE**RRRR**A-), a triple arginine (RRR) carries ER accumulation information [29]. Finally, a comparison of the GCSI sequences available has shown that di-arginine motifs at the N-terminal end of these ER resident proteins are highly conserved (Table 1) [42,59].

In mammals, arginine-rich or di-lysine ER-localization signals require a strict spacing relative to the N/C terminus and from the membrane. [22,28,60]. This could also explain why, too close to the transmembrane domain of *At*GCSI, the RR motif at position 21,22 does not confer ER localization (Table 1). A similar situation was described when a deleted version of *A. thaliana *mannosidase II (ManII) containing a 10 aa cytosolic tail (MP**RKR**TLVVN-) was targeted to the Golgi only, despite an RXR motif in the sequence [61,62]. These examples suggest that position of the di-arginine motif(s) relative to the N-terminal end and/or the TMD is certainly important to consider in plants too.

Interestingly, in mammalian cells, in contrast to KK-signals, functional arginine-rich signals are found in a variety of cytosolic positions, including intracellular loops and the N- and C- termini in type II and type I membrane proteins, respectively [28,46]. Here, we have identified a sequence similar to the GCSI arginine-rich sequence, in the C-terminal cysosolic tail of the type I membrane protein *A. thaliana *calnexin (ND**RR**PQ**R**X**R**PA-) [63] and we have shown that this sequence has the capacity to relocate a type II Golgi protein to the ER. These results are consistent with previous data showing that the last 78 C-terminal aa of calnexin, including a 43 aa CT, a 22 aa TMD and 13 aa in the lumen, were sufficient to target GFP to the ER [64]. All together these results suggest that cytosolic arginine-rich motifs might have a similar role for residency of type II and some type I ER membrane proteins in the ER of plant cells.

### The luminal domain of *At*GCSI also contains ER targeting information

While performing successive deletions in order to identify a minimal ER targeting sequence in *At*GCSI, we have observed that when the 13 N-terminal aa were removed from the full length protein, Δ13GCSI was still located in the ER. This result clearly shows that the arginine-rich cytosolic tail is not the only ER determinant in *At*GCSI. A series of deletions at the C-terminal end of Δ13GCSI led us to identify a luminal sequence containing ER targeting information. When fused to XYLT35, a 60 aa luminal sequence from Pro91 to Cys150 of *At*GCSI is able to almost perfectly relocate this medial Golgi marker into the ER. This is the first time that an ER localization signal is shown to be contained in the luminal domain of a plant membrane protein. As mentioned above, the Golgi labeling occasionally observed with this fusion protein might be due to a competition between the ER localization sequence from *At*GCSI and the TMD length of XYLT35 more adapted to Golgi than ER location.

As shown here for *At*GCSI, some mammalian and yeast membrane proteins also contain two ER retention/retrieval signals [18,65,66]. For instance, in human GCSI, the CT bears a triple-arginine ER-targeting motif and the luminal domain contains an ER retention domain which is yet to be characterized [29]. In conclusion, at least for ER resident membrane proteins, the presence of several sequences containing ER targeting information seems to be common. Interestingly, different motifs also probably suggest a hierarchy of these signals and different targeting mechanisms and the importance for those proteins to be kept securely in the ER.

### Several mechanisms participate to *At*GCSI retention in the ER

In mammalian cells, studies have shown that both retrieval and retention mechanisms govern the localization of ER membrane proteins [11]. Of these two mechanisms, retrieval is better understood, and retrieval signals have been identified in the cytosolic tails of type I and type II ER resident membrane proteins [11,32,67]. In plants, very few data are available on retrieval of ER membrane proteins. Contreras et *al*. [31,68] have shown that a KK motif in the C-terminal cytoplasmic tail of type I p24 protein is able to interact with components of the COPI machinery and to recruit ARF1 *in vitro*. McCartney et *al*. [14] have highlighted a dominant negative mutant of ARF1 affect the transient localisation in the Golgi of a chimera protein containing a -YNNKL motif in its cytoplasmic tail. However, mechanisms by which membrane proteins containing an arginine motif are targeted to the ER remain to be investigated. GCS90 and derivated constructs appear as excellent tools to study these mechanisms in plant cell.

The situation is complicated by the fact that retrieval mediated by arginine or lysine-motifs involves distinct machinery. For instance, a mammalian α-COPI isoform interacts with the KKXX motif but not with the RXR motif [69] and there is also evidence suggesting a COPI-independent ER retrieval pathway [70]. On the other hand, some membrane proteins, such as the type II membrane protein Sec12p are retrieved by interaction of their TMD with the receptor rer1p [33,71,72]. Thus, the questions concerning distinct protein sorting machineries and/or mechanisms for the different ER retrieval motifs remain to be addressed.

In addition to retrieval, a mechanism of retention *sensu stricto *has been described, especially for soluble ER residents. Indeed, it is now generally accepted that soluble reticuloplasmins are retained in the ER lumen of mammalian cells mainly by a mechanism of strict retention. However, when they escape this first mechanism, the ER resident proteins are retrotransported from the Golgi back to the ER by a second mechanism involving a H/KDEL C-terminal sequence and a membrane receptor named ERD2. Some data are also in favor of the presence of these two mechanisms to explain retention of reticuloplasmins in the plant ER [9].

Although one could argue that ER retention is due to the absence of positive signals required for an efficient ER exit [73], it is likely that specific retention signals or features are also necessary to prevent massive access of ER-resident membrane proteins into forward carriers [65,74].

ER retention of type I and type II membrane proteins can be accomplished by direct association of protein subunits to give large oligomeric complexes *via *their TMD and/or luminal domain, as previously described in the kin-recognition model for Golgi-located membrane proteins [34,38,75-77]. This type of mechanism may be functional in the ER retention of subunit components of the hetero-oligomeric oligosaccharyltransferase complex [78,79]. When expressed in COS cells, ER targeting information in the luminal domain of human GCSI appears to direct ER localization by retention rather than by retrieval. Evidence includes the fact that N-linked Man_9_-GlcNAc_2 _is the major glycan released from the recombinant enzyme [29]. On the other hand, the co-purification of α-glucosidase I from either bovine mammary glands or calf liver with a large 320-350 kDa protein complex is consistent with homotetramer formation responsible for ER retention [80,81].

**Figure 10:**
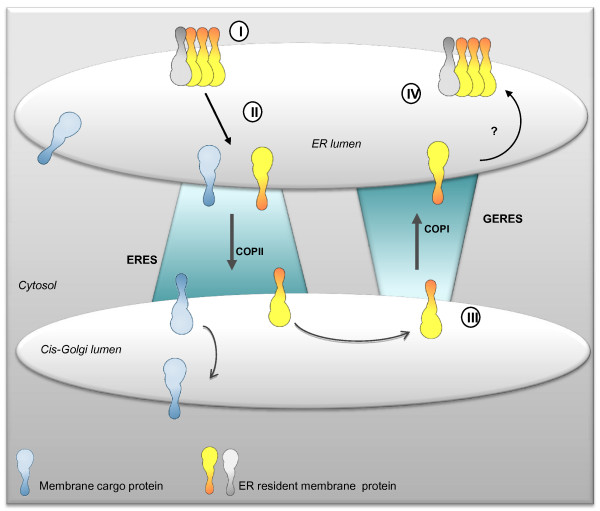
**Schematic representation of mechanisms involved in the location of type II membrane protein in the plant ER**. Two mechanisms for ER localization of GCSI are proposed, one being complementary of the other. First, *At*GCSI resides in ER subdomains where it forms homo or hetero-oligomers with an unknown partner and is excluded from the ER export sites (ERES) (**I**). When *At*GCSI molecules escape these complexes, they move to the ERES (**II**) and are transported from the ER to the Golgi in a COPII dependent manner. Once in the Golgi, the COPI machinery would recognize *At*GCSI's cytosolic tail (**III**). Retrograde transport would then occur at Golgi-ER export sites (GERES) to target *At*GCSI back to the ER where it would form new complexes with its partners (**IV**).

Although there is now evidence that both protein retrieval and retention mechanisms operate at the ER-Golgi interface, the question concerning the relative roles played by these different mechanisms in determining the residency of ER membrane proteins is still largely unresolved. The following targeting model could be put forward for *At*GCSI. We have shown that, at least, two ER localization signals are present in *At*GCSI and we propose that these signals correspond to different targeting mechanisms. As illustrated Figure 10, *At*GCSI would form homo- or heterooligomers (*via *the luminal region) that are excluded from ER domains where ERES are formed. When *At*GCSI monomers escaping these large complexes, are transported by default simultaneously with membrane proteins containing export signals to the Golgi *via *a COPII-mediated transport, *At*GCSI molecules arriving in the *cis*-Golgi interact with putative di-arginine specific receptors mediating their COPI-dependent retrotransport to the ER. The presence of punctate structures in some of the GCSI mutants is also in favor of an arginine-based retrieval.

### Preliminary evidence for Golgi-ER exit sites (GERES)

In contrast to mammalian cells, transport of proteins in plants between the ER and the Golgi does not rely on the cytoskeleton but nevertheless requires energy and is regulated by various proteins such as the GTPases Sar1 and ARF1 [39,41,58,82]. Forward transport of proteins is initiated in specific regions of the ER membrane called ERES (ER Exit Sites) that were visualized using fluorescent protein fusions to plant homologues of the proteins involved in the COPII-coat formation in mammalian cells, for instance Sar1 [48], Sec23 [52,83], Sec24 [83,84] and Sec13 [52]. Very recently, ERES were shown to be induced not only by membrane cargo but also by specific exit sequences [84]. Regarding *At*GCSI, when one out of the three di-arginine motifs is present in the cytosolic tail of a GCS90, fluorescence is detected not only in the ER but also in punctate structures close to- and moving with the Golgi stacks along ER tracks. Both soluble and membrane ER markers are excluded from these punctate structures. We propose that they correspond to a Golgi/ER intermediate compartment. Interestingly, we have shown that punctate structures do not colocalize with Sar1 WT and are not observed in the presence of Sar1p-GTP. However, as expected, Sar1p-GTP-mRFP exerts a dominant negative effect on protein secretion and retains the Golgi construct R/LGCS90 in the ER, showing that ER exit of R/LGCS90 is COPII-regulated, as it was previously shown for many other membrane or soluble proteins [48-51]. Moreover, in our expression system, punctate structures disappeared in the presence of BFA. In the same way, BFA prevented cargo-induced recruitment of Sar1p-YFP at the ERES (ERD2-GFP being the cargo) [48]. It remains to be elucidated whether is due to the loss of Golgi stacks or blockage of ER exit sites, the fact that punctate structures could not be seen after BFA treatment. However, these results strongly support that the punctate structures are involved in Golgi to ER traffic, therefore we propose that those structures that do not colocalize with Sar1 as described for ERES, could well be Golgi-ER Export Sites (GERES).

## Conclusion

*Arabidopsis *alpha glucosidase I (*At*GCSI) is the first enzyme involved in the N-glycan maturation. We have previously shown that the function and consequently the location of this type II membrane protein in the ER is essential for *Arabidopsis *development [42].

As illustrated Figure 10, we have identified two independent types of signals conferring ER residency in the *At*GCSI sequence. Three distinct di-arginine motifs co-existing in the cytosolic tail of *At*GCSI and a 60 aa luminal sequence are independently sufficient for ER retention. Interestingly, the presence of these different types of signals suggests that both retrieval and retention mechanisms govern the localization of *At*GCSI in the ER membrane. When only one out of the three di-arginine motifs is present, *At*GCSI accumulates not only in the ER but also in punctate structures not yet characterised at the ER/Golgi interface and tentatively named GERES. We hypothesised that GERES correspond to Golgi to ER export sites involved at least in arginine-based retrieval mechanisms from the Golgi back to the ER.

## Methods

### Glucosidase I-GFP fusions

The binary vector pBLTI121-sGFP was generated by inserting cDNA encoding sGFP without the ATG [85] as a *Spe*I and *Stu*I fragment into the binary plant transformation vector pBLTI121 [9]. The full length *At*GCSI cDNA was amplified by polymerase chain reaction (PCR) using forward primer FGCSI (CGGGGTACCCCATGACCGGAGCTAGCCGT) and reverse primer RGCSI (CGGGATCCGAAAAATAGGATAATCTTC) and sub-cloned into pBLTI121-GFP as a *Kpn*I or *BamH*I fragment.

The different glucosidase-GFP fusions were then generated by PCR using the *At*GCSI as template and were all fused at the N-terminal end of GFP using *Kpn*I and *Spe*I restriction sites into pBLTI121-GFP. Thirteen different GFP fusions were made. They are schematized in Figure 1 and Table 2 and the primers used are detailed in Table 3. GSC150 and GCS90 correspond to the first 150 and 90 aa of *At*GCSI respectively fused to GFP. Δ13GCS90 and Δ13GCS150 derivate from GCS150 and GCS90, respectively, where the first 13 aa were deleted. Directed mutagenesis led to the replacement of the arginine residues located at the position 6, 7, 10 and/or 12 with leucine or alanine residues and the constructs were named R/LGCS90-GFP, R/AGCS90-GFP, R/L_6-7_GCS90-GFP, R/L_10-12_GCS90-GFP, R/L_6-10_GCS90-GFP, R/L_6-12_GCS90-GFP, R/L_7-10_GCS90-GFP and R/L_7-12_GCS90-GFP.

**Table 3:** Oligonucleotides used to generate GFP fusions.

Primer	5'-3' sequence
	At glucosidase I as template
RGCS150	GACTAGTACACAAATGCCGCATAAC
RGCS90	GACTAGTAAAAGGAGTGATAACCCT
FΔ13GCS90/150	CGGGGTACCCCATGAAATCATCATCATTATCTCCC
FR/L4	CGGGGTACCCCATGACCGGAGCTAGC**CTTCTG**AGCGCG**CTT**GGT**CTA**ATCAAATCATCA
FR/A4	CGGGGTACCCCATGACCGGAGCTAGC**GCTGCG**AGCGCG**GCT**GGT**GCA**ATCAAATCATCA
FR/L6-7	CGGGGTACCCCATGACCGGAGCTAGC**CTTCTG**AGCGCGCGT
FR/L10L12	CGGGGTACCCCATGACCGGAGCTAGCCGTCGGAGCGCG**CTT**GGT**CTA**ATCAAATCATCA
FR/L6L10	CGGGGTACCCCATGACCGGAGCTAGC**CTT**CGGAGCGCG**CTT**GGTCGAATCAAATCATCA
FR/L6L12	CGGGGTACCCCATGACCGGAGCTAGC**CTT**CGGAGCGCGCGTGGT**CTA**ATCAAATCATCA
FR/L7L10	CGGGGTACCCCATGACCGGAGCTAGCCGT**CTG**AGCGCG**CTT**GGTCGAATCAAATCATCA
FR/L7L12	CGGGGTACCCCATGACCGGAGCTAGCCGT**CTG**AGCGCGCGTGGT**CTA**ATCAAATCATCA
Fhs10GCS90	CGGGGTACCCCATGGCTCGGGGCGAGCGGCGGCGCCGCGCAAA
FGCS(70-150)	GGGGTACCCGGCTAGTTCGTCACGGG
FGCS(91-150)	GGGGTACCCCTGCTCCGAAAGTCATG

	β1,2xylosyltransferase as template
FGCS13XYLT35	CGGGGTACCCCATGACCGGAGCTAGCCGTCGGAGCGCGCGTGGTCGAATCAGTAAACGGAATCCGAAG
FCNX11XYLT35	CGGGGTACCCCATGAATGATCGTAGACCGCAAAGGAAACGCCCAAGTAAACGGAATCCGAAG-3'
RXYLT35	GGACTAGTTGAAAACGACGATGAGTG
FXYLT35'	GCTCTAGAGCATGAGTAAACGGAATCCG
RXYLT35'	GGGGTACCTGAAAACGACGATGAGTG

The *BamH*I, *Spe*I or *Kpn*I restriction sites used for vector construction are underlined. Triplet codons for leucine or alanine are given in bold.

Finally, the N-terminal 13 aa of *At*GCSI were replaced by the N-terminal 10 aa of human hippocampus glucosidase I [86] to obtain hs10GCS90-GFP. The reverse primer RGCS150 was used to generate GCS150-GFP and Δ13GCS150, and the oligonucleotide RGCS90 was common to all the other fusions ending at aa 90.

### β-1,2-xylosyltransferase-derivated GFP fusions

To obtain the fusion protein GCS13-XYLT35 or CNX11-XYLT35, nucleotides coding for the first N-terminal 13 aa of *At*GCSI or last C-terminal 11 aa of *A. thaliana *calnexin were fused to the 5' end of XYLT35 after PCR amplification and using XYLT35 cDNA as template [47]. Primers contain respectively a *Kpn*I or a *Spe*I site (underlined, Table 3) to permit the cloning into pBLTI121-GFP.

Two cDNAs encoding 81 and 60 aa of the luminal predicted domain of *At*GCSI were fused to the 3' end of XYLT35. To generate the fusion proteins XYLT35-GCS*lum81 *and XYLT35-GCS*lum60*, the first N-terminal 35 aa of XYLT were first subcloned into pBLTI121-GFP, at the N-terminal end of GFP. Then, XYLT35 was amplified by PCR using primers FXYLT35' and RXYLT35' detailed in table 3, and a *Xba*I or *Kpn*I restriction site (underlined) was used to clone XYLT35 into pBLTI121-GFP. Finally, the 81 or 60 aa of predicted luminal domain of *At*GCSI, aa 70 to aa 150 or aa 91 to 150, were subcloned between XYLT35 and GFP using PCR reaction with *At*GCSI cDNA as template and forward primer FGCS80 or FGCS60 (see table 2 for primer details) and reverse primer R150 (see above) with respectively *Kpn*I or *BamH*I site (underlined) to sub-clone into pBLTI121.

### ER and Golgi red fluorescent markers

Monomeric red fluorescent protein (mRFP) was cloned in pCAMBIA binary vector under the control of sporamine signal peptide at the 5'end and the ER targeting sequence HDEL at the 3' end. ST-mRFP, described in Saint-Jore-Dupas et *al*., [44] was amplified by PCR using forward primer FST and reverse primer RST (Table 3) and sub-cloned into pCAMBIA as a *Kpn*I or *SacI *fragment. For the XYLT35mRFP, MAN99mRFP and the GCS90mRFP, we have then substituted the GFP from the XYLT35, MAN99 and GCS90 constructs by the mRFP using the *Spe*I and *Sac*I endonucleases.

### *Agrobacterium*-mediated tobacco BY-2 cell transformation

pBLTI121-GFP fusions were transferred into *Agrobacterium tumefaciens *(strain LBA4404) by heat shock [87]. Transgenic *Agrobacterium *were selected onto YEB medium (per liter, beef extract 5 g, yeast extract 1 g, sucrose 5 g, MgSO4-7H2O 0.5 g) containing kanamycin (100 mg.mL^-1^) and gentamycin (10 mg.mL^-1^) and were used to transform *Nicotiana tabacum *(*c.v. Bright Yellow-2*) BY-2 cells, as described in Gomord et *al*., [88]. Transformed tobacco cells were selected in the presence of cefotaxime (250 mg.mL^-1^) and kanamycin (100 mg.mL^-1^). After screening by fluorescence microscopy and, calli expressing the GFP fusions were used to initiate suspension cultures of transgenic cells.

### *Agrobacterium*-mediated transient expression in *Nicotiana tabacum*

PBLTI121-GFP, pVKH18-En6-mRFP, Sar binary plasmid and pCAMBIA-mRFP fusions transformed *A. tumefaciens *(strain GV3101 pMP90) [89] were cultured in kanamycin/spectinomycin and gentamycin containing YEB at 28°C until the stationary phase (approximately 20 h), washed and resuspended in infiltration medium (MES 50 mM pH5.6, glucose 0.5%(w/v), Na3PO4 2 mM, acetosyringone (Aldrich) 100 mM from 10 mM stock in absolute ethanol. The bacterial suspension was pressure injected into the abaxial epidermis of plant leaves using a 1-mL plastic syringe by pressing the nozzle against the lower leaf epidermis. Plants were incubated for 2-3 days at 20-25°C [58].

### BFA treatment

Tobacco cells were incubated in 50 μm.mL^-1 ^BFA (Sigma, from 10 mg.mL^-1 ^stock in DMSO) for 2 h before confocal analysis as described in [58].

### Confocal Laser Scanning Microscopy analysis

Cells expressing GFP were imaged using a Leica TCS SP2 AOBS confocal laser scanning microscope (CLSM) with a 488-nm argon ion laser line and the fluorescence was recorded by a photomultiplier set up for 493-538 nm. Dual-color imaging of cells co-expressing GFP and mRFP was performed using simultaneously a 488-nm argon ion laser line with the lowest laser power and a HeNe 594 nm laser line. Fluorescence signals were separated using the acousto-optical beam splitter (AOBS) and GFP emission was detected in photomultiplier 2 (493-538 nm) whereas mRFP was collected in photomultiplier 3 (600-630 nm). Appropriate controls were performed to exclude the possibility of cross talk between the two fluorophores before the image acquisitions.

### Accession numbers

[EMBL: Z18242 (*A. thaliana *calnexin; Huang et *al*., 1993); EMBL: X87237 (*H. sapiens *glucosidase I; Kalz-Füller et *al*., 1995); EMBL:AJ278990 (A. *thaliana *glucosidase I; Boisson et *al*., 2001); EMBL:AF272852 (A. *thaliana β*-1,2-xylosyltransferase; Pagny et *al*., 2003)].

## List of abbreviations

CD: C-terminal domain; CT: cytosolic tail; ER: endoplasmic reticulum; GCS: glucosidase; GFP: green fluorescent protein; MAN: mannosidase; mRFP: monomeric red fluorescent protein; ST: sialyltransferase; TMD: transmenbrane domain; XYLT: xylosyltransferase.

## Authors' contributions

AB carried out the molecular genetic studies, and made a substantial contribution to the confocal microscopy analysis and interpretation of data. CSJD made a substantial contribution to the confocal microscopy analysis and interpretation of data. MCHG, SPS, CP, FG, MCKM and VG carried out the molecular genetic studies, and made contributions to construct design. AB, CSJD, CR, LF and VG have been involved in drafting the manuscript or revising it critically for important intellectual content. GV has given final approval of the version to be published. All authors read and approved the final manuscript.

## Supplementary Material

Additional file 1 **ER membrane protein biosynthesis and topology**. (**A**) Type II membrane proteins are synthesized with an internal start-transfer sequence that is blocked in the membrane during the translation of the protein in the ER lumen. (**B**) In contrast, type I membrane proteins are synthesized with a cleavable hydrophobic signal peptide at their N-terminal ends for introduction in the ER (similar to what happens to a soluble protein) and a stop transfer sequence that corresponds to the transmembrane domain. Click here for file

Additional file 2 **The arginine-rich cytosolic domain of type I calnexin targets the type II Golgi marker XYLT35 to the ER when fused at its N-terminal end**. (**A**)* Arabidopsis thaliana *calnexin (a type I membrane protein) contains a C-terminal cytosolic, 11 amino acid long-, arginine-rich-peptide that has never been characterized especially for targeting efficiency (yellow rectangle). This RRXXRXR peptide is very similar to the one found at the cytosolic N-terminal end of type II *A. thaliana *glucosidase I. (**B**) To determine if the arginine-rich motif from calnexin could mediate the targeting of a type II membrane protein in the ER, it was fused to the N-terminal end of the Golgi marker XYLT35 (CNX11-XYLT35, Table 2). When transiently expressed in tobacco leaf epidermal cells, CNX11-XYLT35 (left) was found mainly in the ER (middle) and in part in the Golgi (right), exactly as observed for GCS13-XYLT35 (Figure 3G-I). Bars = 8 μm. Click here for file

Additional file 3 **The spacing between arginine residues is important to confer ER retention**. CLSM analysis of *Nicotiana tabacum *leaf epidermal cells co-expressing GFP-fusions together with either the ER marker mRFP-HDEL (left panel) or the Golgi marker ST-mRFP (right panel). R/L_6-12_GCS90 is located to the ER and to punctate structures that do not contain the ER soluble protein mRFP-HDEL (**A**) and are closely associated to the Golgi stacks (**B**). In contrast, R/L_6-10_GCS90 and R/L_7-12_GCS90 colocalize with mRFP-HDEL (**C **and **E **respectively) and with ST-mRFP (**D **and **F **respectively). These data show that the LRXXLXR and RLXXRXL motifs are not efficient to target GCS90 to the ER exclusively. Finally, R/L_7-10_GCS90 is found exclusively in the Golgi (**H**) and not in the ER (**G**). In conclusion, arginine residue spacing and their position relative to the N-terminal end are important for ER targeting efficiency. Bars = 8 μm. Click here for file

Additional file 4 **Punctate structures and Golgi move together along the ER**. Tobacco leaf epidermal cells coexpressing R/L_6-7_GCS90-GFP and XYLT35-mRFP. Click here for file
