# Supplementary material for: Cytosolic N-terminal arginine-based signals together with a luminal signal target a type II membrane protein to the plant ER
Source: BMC Plant Biol. 2009 Dec 8;9:144. doi: 10.1186/1471-2229-9-144 (PMC2799409; doi:10.1186/1471-2229-9-144)
Supplement: Additional file 1 — ER membrane protein biosynthesis and topology. (A) Type II membrane proteins are synthesized with an internal start-transfer sequence that is blocked in the membrane during the translation of the protein in the ER lumen. (B) In contrast, type I membrane proteins are synthesized with a cleavable hydrophobic signal peptide at their N-terminal ends for introduction in the ER (similar to what happens to a soluble protein) and a stop transfer sequence that corresponds to the transmembrane domain. [file 1471-2229-9-144-S1.PPT]

## Slide 1
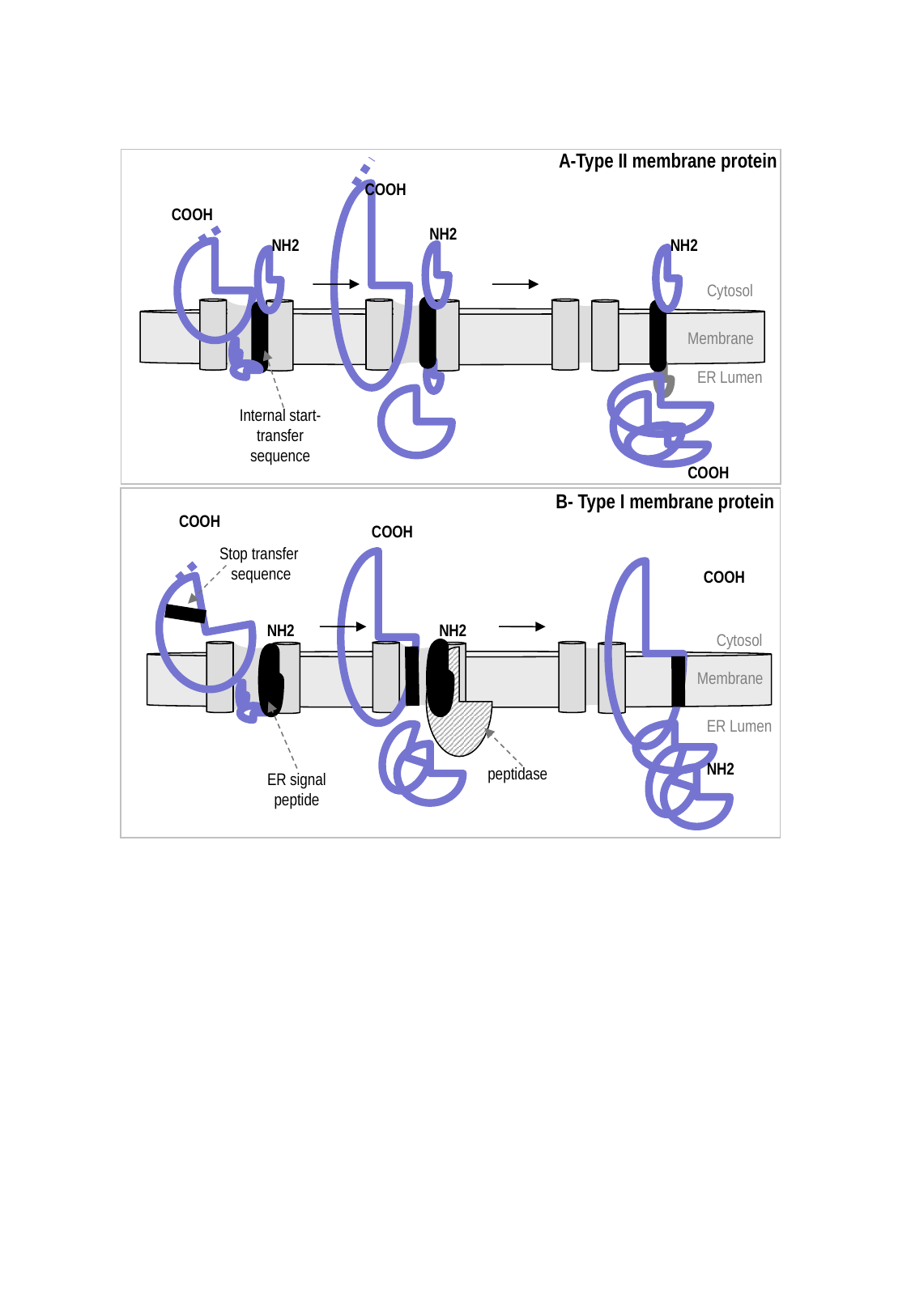

A-Type II membrane protein
COOH
COOH
NH2
NH2
NH2
Cytosol
Membrane
ER Lumen
Internal start-transfer sequence
COOH
B- Type I membrane protein
COOH
COOH
Stop transfer
sequence
COOH
NH2
NH2
Cytosol
Membrane
ER Lumen
NH2
peptidase
ER signal peptide
