# Supplementary material for: Cytosolic N-terminal arginine-based signals together with a luminal signal target a type II membrane protein to the plant ER
Source: BMC Plant Biol. 2009 Dec 8;9:144. doi: 10.1186/1471-2229-9-144 (PMC2799409; doi:10.1186/1471-2229-9-144)
Supplement: Additional file 2 — The arginine-rich cytosolic domain of type I calnexin targets the type II Golgi marker XYLT35 to the ER when fused at its N-terminal end. (A) Arabidopsis thaliana calnexin (a type I membrane protein) contains a C-terminal cytosolic, 11 amino acid long-, arginine-rich-peptide that has never been characterized especially for targeting efficiency (yellow rectangle). This RRXXRXR peptide is very similar to the one found at the cytosolic N-terminal end of type II A. thaliana glucosidase I. (B) To determine if the arginine-rich motif from calnexin could mediate the targeting of a type II membrane protein in the ER, it was fused to the N-terminal end of the Golgi marker XYLT35 (CNX11-XYLT35, Table 2). When transiently expressed in tobacco leaf epidermal cells, CNX11-XYLT35 (left) was found mainly in the ER (middle) and in part in the Golgi (right), exactly as observed for GCS13-XYLT35 (Figure 3G-I). Bars = 8 μm. [file 1471-2229-9-144-S2.PPT]

## Slide 1
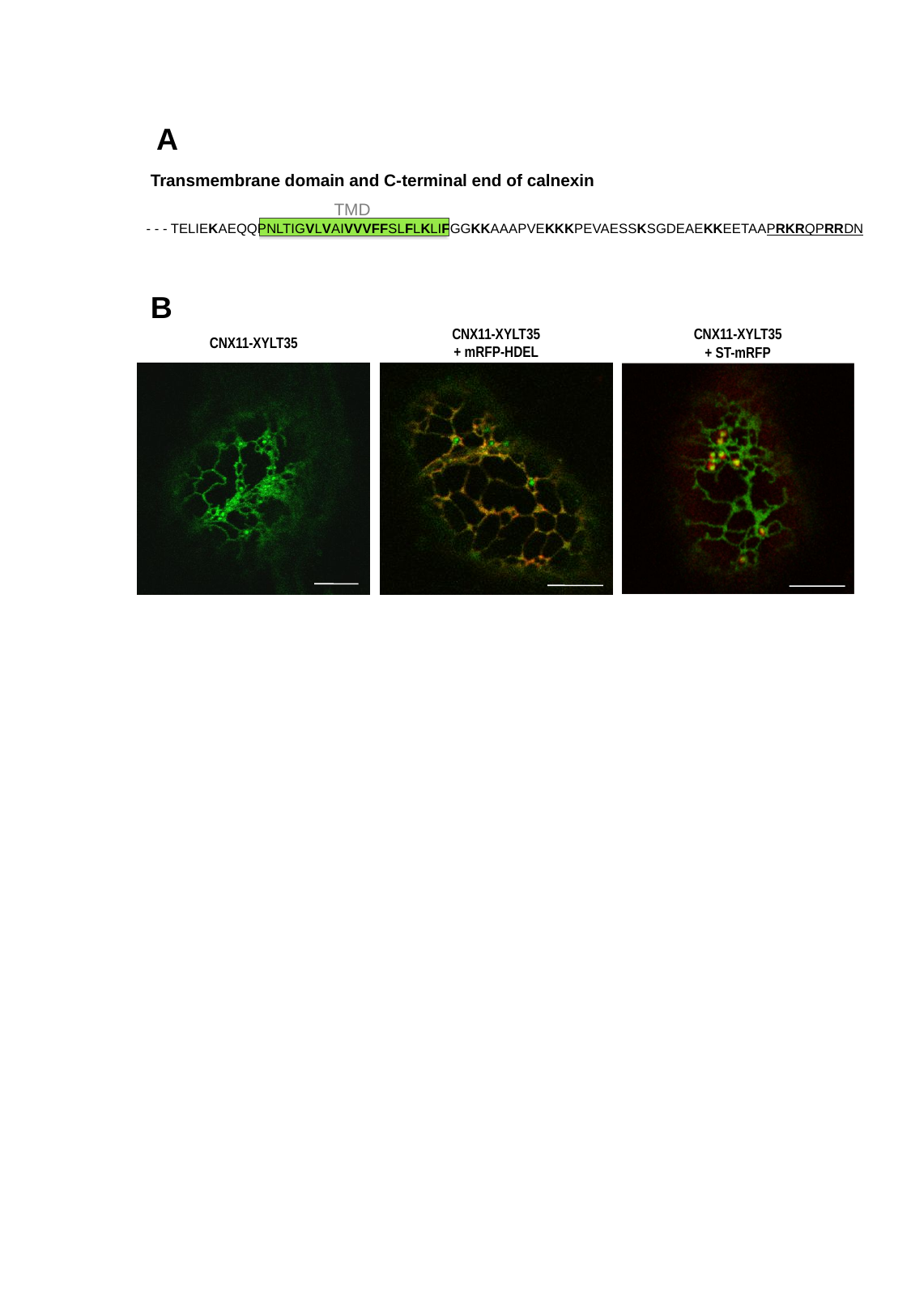

A
Transmembrane domain and C-terminal end of calnexin
TMD
- - - TELIEKAEQQPNLTIGVLVAIVVVFFSLFLKLIFGGKKAAAPVEKKKPEVAESSKSGDEAEKKEETAAPRKRQPRRDN
B
CNX11-XYLT35
+ ST-mRFP
CNX11-XYLT35
CNX11-XYLT35
+ mRFP-HDEL
