# Supplementary material for: Cytosolic N-terminal arginine-based signals together with a luminal signal target a type II membrane protein to the plant ER
Source: BMC Plant Biol. 2009 Dec 8;9:144. doi: 10.1186/1471-2229-9-144 (PMC2799409; doi:10.1186/1471-2229-9-144)
Supplement: Additional file 3 — The spacing between arginine residues is important to confer ER retention. CLSM analysis of Nicotiana tabacum leaf epidermal cells co-expressing GFP-fusions together with either the ER marker mRFP-HDEL (left panel) or the Golgi marker ST-mRFP (right panel). R/L6-12GCS90 is located to the ER and to punctate structures that do not contain the ER soluble protein mRFP-HDEL (A) and are closely associated to the Golgi stacks (B). In contrast, R/L6-10GCS90 and R/L7-12GCS90 colocalize with mRFP-HDEL (C and E respectively) and with ST-mRFP (D and F respectively). These data show that the LRXXLXR and RLXXRXL motifs are not efficient to target GCS90 to the ER exclusively. Finally, R/L7-10GCS90 is found exclusively in the Golgi (H) and not in the ER (G). In conclusion, arginine residue spacing and their position relative to the N-terminal end are important for ER targeting efficiency. Bars = 8 μm. [file 1471-2229-9-144-S3.PPT]

## Slide 1
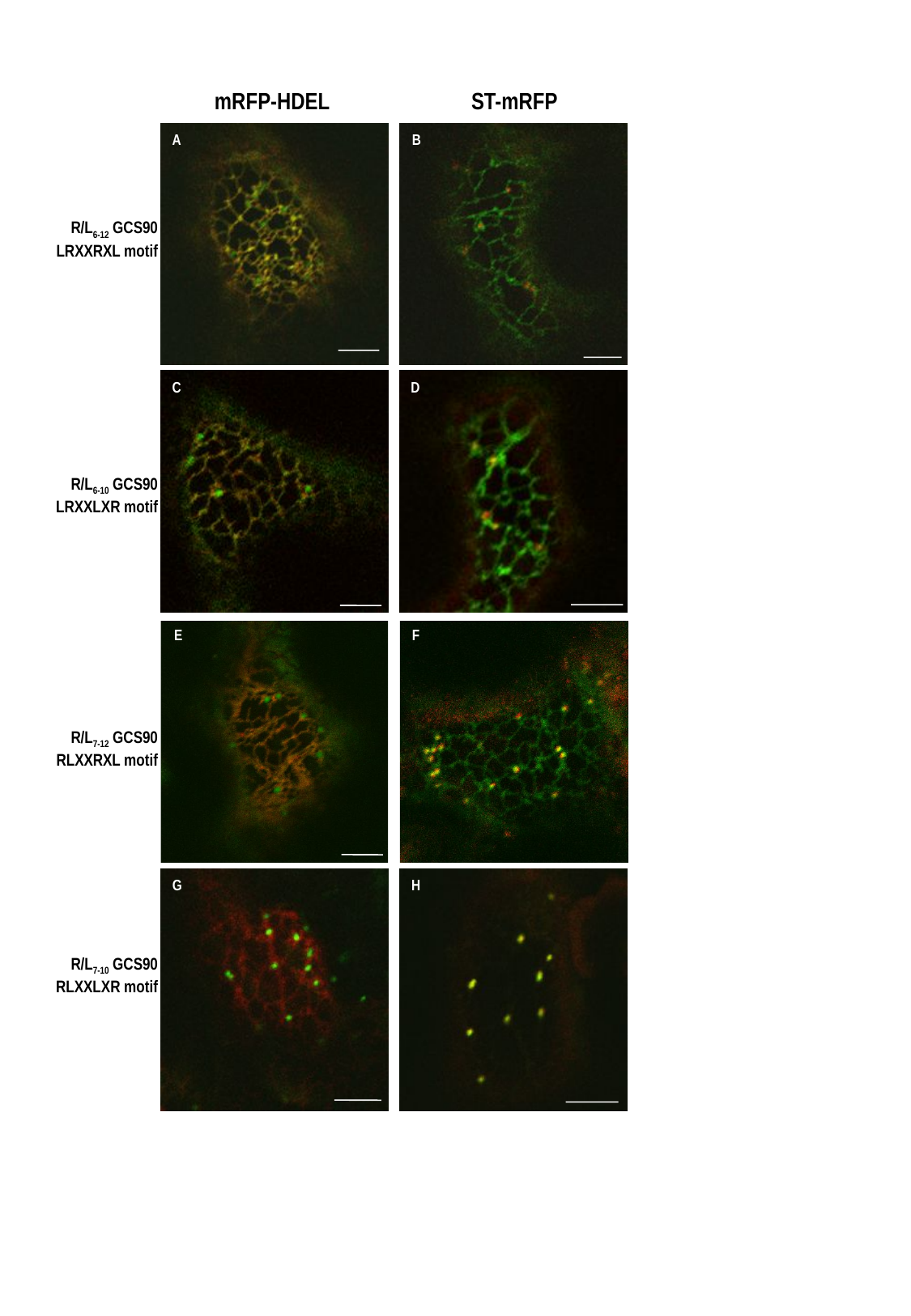

ST-mRFP
mRFP-HDEL
A
B
R/L6-12 GCS90
LRXXRXL motif
C
D
R/L6-10 GCS90
LRXXLXR motif
E
F
R/L7-12 GCS90
RLXXRXL motif
G
H
R/L7-10 GCS90
RLXXLXR motif
